# Post-Inflammatory Hypopigmentation: Review of the Etiology, Clinical Manifestations, and Treatment Options

**DOI:** 10.3390/jcm12031243

**Published:** 2023-02-03

**Authors:** Medha Rao, Katherine Young, Ladonya Jackson-Cowan, Arianne Kourosh, Nicholas Theodosakis

**Affiliations:** 1 Department of Dermatology, Massachusetts General Hospital, Boston, MA 02114, USA; 2 Robert Wood Johnson Medical School, New Brunswick, NJ 08901, USA; 3 Harvard Medical School, Boston, MA 02214, USA; 4 The Medical College of Georgia at Augusta University, AU/UGA Medical Partnership, Athens, GA 30602, USA

**Keywords:** post-inflammatory, hypopigmentation, inflammation, pityriasis alba, lichen striatus, pityriasis lichenoides, lichen sclerosus, progressive macular hypomelanosis, discoid lupus, pityriasis versicolor, scleroderma, mycosis fungoides, leukoderma syphiliticum, acrodermatitis chronica atrophicans, iatrogenic, laser, ultraviolet, corticosteroids

## Abstract

Post-inflammatory hypopigmentation is a common acquired pigmentary disorder that is more prominent in skin of color, leading to great cosmetic and psychosocial implications. Often, a diagnosis with a pigmentary disorder can negatively impact an individual’s health-related quality of life and may result in stigma. Although most cases of post-inflammatory hypopigmentation resolve spontaneously over time, a systematic diagnostic approach can help with identifying the underlying etiology and informing treatment strategies. It can be due to cutaneous inflammation, sequelae of inflammatory or infectious dermatoses, or dermatologic procedures. Therefore, a thorough understanding of the epidemiology, patient history, physical exam findings, and clinical features of post-inflammatory hypopigmentation phenomenon can explain the primary cause to providers and allow for patient education. It is also important to understand the various therapeutic approaches available and the efficacy of these options, which will inform providers to choose the appropriate therapy for patients. Although algorithms exist for classifying acquired disorders of hypopigmentation, there are no established algorithms for the diagnosis and treatment of post-inflammatory hypopigmentation, which warrants further exploration and discourse.

## 1. Introduction

Post-inflammatory hypopigmentation (PIH) is a common acquired pigmentary disorder that is more prominent in skin of color. It represents an acquired partial or total loss of skin pigmentation due to cutaneous inflammation, sequelae of inflammatory or infectious dermatoses, or dermatologic procedures [1]. Proposed mechanisms for PIH include decreased melanin production, blocked transfer of melanosomes to keratinocytes, and melanocyte death [2]. Variations in cutaneous manifestation in response to trauma or inflammatory insults can be explained by an individual’s chromatic tendency, possibly an inheritable trait [3]. It is thought those with robust melanocytes react with increased melanin production, presenting clinically as hyperpigmentation. In contrast, less robust melanocytes are susceptible to damage resulting in hypopigmentation [3]. Nevertheless, other factors, such as severity, duration, and type of inflammation, may also play a role.

Social stigma surrounding dyspigmentation can impact an individual’s self-esteem, often negatively affecting their quality of life [4]. Although no study has looked at the prevalence and psychological burden of PIH specifically, pigmentary disorders (i.e., melasma, vitiligo acquired dermal macular hyperpigmentation) have been associated with increased risk of anxiety, depression, and somatoform disorders [5]. The total healthcare cost and utilization is unknown, but disorders of pigmentation are the third most common reason for skin of color patients to seek dermatological treatment [6]. Between 1993 and 2010, an estimated 24.7 million outpatient visits were made related to dyschromia [7]. Despite the prevalence of dyspigmentation especially in skin of color individuals, treatment options remain limited. In this narrative review, we explore the etiology, clinical manifestations, and treatments in the context of PIH and provide insight into diagnostic methodology.

## 2. Methods

We conducted a review of the literature utilizing PubMed and Google Scholar databases to provide an up-to-date overview of the research on conditions that cause post-inflammatory hypopigmentation from the dermatologist’s perspective. Databases were searched for relevant articles from database inception to the time of search (December 2022). The results are presented in the form of a topical review in which the selection of the content is based on the authors’ experience.

## 3. Pityriasis Alba

### 3.1. Etiology

Pityriasis alba (PA) represents a common PIH resulting from a variety of inflammatory pathologies. PA is commonly seen in children with a personal history of atopy at sites of previous eczematous eruptions. A linear correlation between PA and atopic dermatitis has been shown, especially in patients with two or more signs of atopy [8]. Functional abnormalities, such as poor skin hydration and corneocyte morphological changes, further support an inflammatory process involving the epidermis [9]. Over-expression of inflammatory cytokines, such as IFN-y and elevated ROS levels, are thought to prevent maturation of melanosomes, cause abnormal melanosome transport, and induce lysis of keratinocytes, which can affect the transfer of melanosomes from melanocytes to keratinocytes [10,11]. Some studies have reported no change in number of melanocytes, while others have seen a decrease as duration of lesion increases; however, further studies are needed to understand the significance of these findings [12,13,14]. In general, PA is more noticeable in darker skin types. Photo exposure causes surrounding skin to tan more easily, accentuating the hypopigmented skin [8,15]. Differential tanning may be further explained by hyperkeratosis and parakeratosis of the affected epidermis, a common histological finding in PA secondary to its causative inflammatory dermatosis [14]. This alteration of keratinocytes may protect the skin from UV penetration and subsequent tanning, contributing to hypopigmentation [16]. Although controversial, nutritional deficiencies, such as zinc and copper, have also been associated with hypopigmentation seen in PA [17,18]. Zinc and copper are essential co-factors in the enzymatic reactions of the tyrosinase: the rate-limiting enzyme responsible for melanogenesis [19].

### 3.2. Clinical Manifestations

Signs of inflammatory skin disease, such as atopic dermatitis (i.e., erythematous, eczematous plaques in either the extensor or flexural distribution), will be initially present. Upon resolution of the underlying inflammation, multiple, ill-defined, round, or oval shaped hypopigmented macules and patches with fine scaling are seen [10]. The hypopigmentation is more prominent during the spring or summer months due to darkening of the surrounding skin [20,21].

### 3.3. Treatment

Pityriasis alba is a self-limited disease, and patients can be reassured that most cases resolve within one year. The most important strategy for treating PA is to limit the potential triggering factor. Emollient therapy can be effective to treat the coexisting atopic dermatitis [15]. Topical steroids address the underlying inflammation to accelerate repigmentation and reduce symptoms, such as erythema and pruritis [22]. Results of a prospective study illustrated tacrolimus (a calcineurin inhibitor) ointment, calcipotriol (a vitamin D analog) cream, or topical steroids on target lesions twice daily led to reduced hypopigmentation at 8 weeks in PA patients [20,23]. Topical calcineurin inhibitors are preferred for facial lesions, as they do not carry the long-term risks of steroid induced atrophy or hypopigmentation [24,25]. In addition to its immunomodulatory effects, tacrolimus has shown to induce tyrosinase activity and expression, resulting in melanin biosynthesis in vitro [26]. Some studies have suggested that tacrolimus may also promote migration of melanocytes into hypopigmented areas. [26]. Vitamin D analogs are also known to promote melanogenesis by influencing melanocyte maturation and differentiation [27,28]. In children with moderate to severe facial PA, excimer laser was found to be an effective therapeutic modality to improve hypopigmented lesions, possibly through stimulation of melanin synthesis through activation of microphthalmia-associated transcription factor (MITF): the master regulator of melanogenesis [29,30]. Similarly, marked improvement of extensive PA of unknown etiology was seen in five out of six patients after initiation of oral photochemotherapy (oral psoralens plus UVA (PUVA) or sun exposure) three times per week [31]. Oral photochemotherapy is thought to induce pigmentation by increasing the activity of melanocytes, number of melanosomes, and transfer of melanosomes to keratinocytes, though future histological studies are needed to confirm these proposed findings [32]. Overall, sun protection and limitation of sun exposure are the most important strategies for preventing darkening of surrounding areas of the skin by lessening the appearance of hypopigmentation.

## 4. Lichen Striatus

### 4.1. Etiology

An individual with a genetic predisposition for lichen striatus is thought to present with the disease after exposure to triggers, such as viral infection, immunization, pregnancy, medications, and trauma [33,34]. A family or personal history of atopic diseases, such as atopic dermatitis, asthma, and allergic rhinitis, may also contribute [35]. The mosaic areas follow the lines of Blaschko, an embryonic migration pathway of keratinocyte precursors. The development of embryologically abnormal keratinocyte clones secondary to somatic mutations is thought to result in T-cell mediated inflammatory reactions when immune tolerance is lost [35,36,37]. This theory is supported by histological findings of CD8+ T cells surrounding necrotic keratinocytes [38]. Although the exact pathogenesis of hypopigmentation in LS is unknown, one proposed explanation ascribes pigment loss to transient disruption of the basal and supra-basal layers of the epidermis by inflammation, interfering with melanocyte and keratinocyte function [35,39,40,41]. The resulting immunological destruction of melanocytes is thought to result in hypopigmentation.

### 4.2. Clinical Manifestations

Lichen striatus begins as a sudden eruption of discrete, pink, red, or skin colored papules, along the lines of Blaschko on the extremities and less frequently on the neck, buttock, or trunk region [34,36]. Lichen striatus albus, seen in darker skin individuals, presents with hypopigmented macules at onset rather than pink colored papules [35]. Papules resolve within 6–24 months, and can leave a transient post-inflammatory hypopigmentation, more visible in darker skin [42]. Hypopigmentation can be the first clinical sign if the inflammatory phase is undetected. Lichen striatus often resolves spontaneously within months to years but PIH can persist for years after lesions remit [34,36].

### 4.3. Treatment

Topical corticosteroids are used for the symptomatic treatment of lichen striatus; however, they have not shown to affect the clinical course of the disease or PIH [35]. Improvement of lesions has been described with topical tacrolimus application. For example, a 6-year-old girl with a 3-month history of lichen striatus saw regression of lesions without residual hypopigmentation in 3 months with tacrolimus ointment application [43]. In a separate patient, facial lichen striatus was successfully treated with 0.1% tacrolimus ointment when applied once or twice daily for 6 weeks [44]. Since destruction of the basal layer occurs with prolonged LS, early initiation of topical calcineurin inhibitors may help to prevent subsequent hypopigmentation [41,43]. Excimer laser treatment has also been shown to be effective for targeted treatment of PIH after lichen striatus [40]. Excimer laser has an immunomodulatory effect and can induce T cell apoptosis [41], which may be partially responsible for treatment response in inflammatory dermatoses, such as lichen striatus. In addition, it can stimulate melanocyte proliferation, migration, and differentiation from hair follicles, allowing repigmentation of affected LS lesions [40].

## 5. Pityriasis Lichenoides Chronica (PLC)

### 5.1. Etiology

Pityriasis lichenoides is an umbrella term that encompasses three conditions: pityriasis lichenoides chronica (PLC), pityriasis lichenoides et varioliformis acuta (PLEVA), and febrile ulceronecrotic Mucha-Habermann disease (a subtype of PLEVA) [45]. PLEVA and PLC represent two ends of the clinical spectrum of a single disease, and as a result, the various diagnoses often overlap. This family of diseases may represent an aberrant cell-mediated response to foreign antigens, such as infections (*Toxoplasma gondii*, Epstein-Barr virus, HIV, parvovirus B19, staphylococci, and Group A strep), vaccines (measles, mumps, rubella, and COVID-19 mRNA), and medications [45,46,47]. PLC can also occur due to T-cell lymphoproliferative processes [46] or with clonal CD4 T cell populations observed in cases of PLC in response to an antigenic trigger [48,49]. Although mechanisms of hypopigmentation seen in PL are not well described, it is proposed that cytotoxic effects of CD8+ T cell on melanocytes results in dysfunction and potential loss [50]. Alterations in the expression of CD117, a receptor on the surface of melanocytes, affects downstream targets, such as MITF, regulator of melanin-related gene expression, ultimately affecting melanogenesis [50,51]. Production of tumor necrosis TNF-α by cytotoxic T cells can also downregulate keratinocyte production of basic fibroblast growth factor (bFGF), which is known to stimulate melanocyte proliferation and migration [50,52]. Histopathologic studies of hypopigmented lesions in patients with PLC showed features of post-inflammatory hypopigmentation (19%), residual PLC (52.4%), and active PLC (28.5%) [50]. Although melanocytes were intact in these lesions, decreased pigmentation was noted in the basal layer.

### 5.2. Clinical Manifestations

The initial presentation of PLC involves the development of asymptomatic, erythematous papules with overlying, adherent, mica-like scale distributed across the trunk and proximal extremities. As PLC in general has an indolent clinical course, the papules will spontaneously flatten and regress with residual hyper- or hypopigmented macules with no scarring [45,53]. PIH is more common in patients with darker skin and can be the predominant initial clinical presentation [10,54]. In a retrospective review of 124 children with pityriasis lichenoides, 91% had post-inflammatory pigmentary changes with hypopigmentation observed in 56% with PLC and 54% with PLEVA [55]. The degree of inflammation explains why some experience PIH while others hyperpigmentation. Severe, acute inflammation can cause destruction of melanocytes, resulting in hypopigmentation, while less severe inflammation can cause melanin incontinence, appearing as hyperpigmentation [56].

### 5.3. Treatment

There is a lack of high-quality randomized control studies on PL treatment, and no consensus exists for dyspigmentation prevention or management. Active treatment of PLC for patients presenting with primarily hypopigmented lesions might be required to control the disease [50]. A systematic review recommends narrowband UVB (nbUVB) phototherapy as the first line of therapy for PLC. It is interesting to note that the pathogenesis of hypopigmented MF (HMF); cytotoxic response to proliferation of malignant T cells; is similar to that of PLC, and nbUVB has been successful in clearance of HMF lesions [57,58,59]. Although there is no evidence in the literature to show improvement of hypopigmentation in PLC with nbUVB, many of the effects of nbUVB, direct cytotoxic effect on clonal T cells and decrease in pro-inflammatory cytokines, make it a promising therapeutic option for hypopigmentation warranting further exploration [60,61]. Second line therapies for PLC include oral erythromycin or low dose methotrexate with or without topical corticosteroids [62]. Topical steroids are commonly used for symptomatic management to reduce the underlying inflammation but are not useful in altering the course of disease for adults and children with PLC [53,56,63]. Antibiotics, such as tetracycline and erythromycin, are recommended for treating or preventing infectious triggers because of their anti-inflammatory properties [53,62]. Methotrexate is reserved for extensive of symptomatic PLC cases not responding to first line therapy [62]. Although not shown to target dyspigmentation in PLC, its anti-TNF-α properties can induce melanocyte-stimulating hormone, which stimulates the growth and development of melanocytes [64]. In psoriatic lesions, TNF-α inhibition led to rapid restoration of pigmentation gene expression [64,65]. These theorized mechanisms need to be further explored in the context of targeted treatments for dyspigmentation in PLC.

## 6. Extragenital Lichen Sclerosus (EGLS)

### 6.1. Etiology

Most cases (85%) of lichen sclerosus (LS) are genital cases, with 15–20% with extragenital manifestation. In 6% of cases of LS, only the extragenital form is seen [66]. Pathogenesis involves oxidative stress and autoimmunity, resulting in disruption of fibroblast and collagen homeostasis [67]. Factors such as genetic predisposition, immune dysfunction, infectious agents, hormonal factors, trauma, and medications are thought to play a role [66]. The exact etiology of hypopigmentation in extragenital lichen sclerosus (EGLS) is unknown and information is extrapolated from literature on genital LS. A postulated mechanism for LS involves autoantibodies to ECM1 (extracellular matrix protein 1) disrupting the normal function of the protein, affecting keratinocyte differentiation, and resulting in atrophic changes of the epidermis [68,69]. This altered keratinocyte biology may prevent transfer of melanosomes from melanocytic dendrites, resulting in hypopigmentation. This was seen in histochemistry staining as decreased melanosome transfer to affected keratinocytes despite normal melanogenesis in adjacent melanocytes [70]. Another theory of hypopigmentation is attributed to the unique cytokine profile (IL-1 α, IL-6, TNF-α, IFN-gamma), which may inhibit melanogenesis via blocking maturation of melanosomes, inhibition of melanin formation, and decreased tyrosinase activity [65,67,71]. Melanocyte loss is also suggested to occur secondary to CD8+ T-cell mediated cytotoxicity [70,72,73].

### 6.2. Clinical Manifestations

LS presents with hypopigmented, opalescent, polygonal papules that coalesce to form plaques with sclerosis and erosions. Extragenital involvement commonly includes the chest, back, abdomen, inframammary region, and proximal extremities [74]. Over time, these lesions will become atrophic with a thin, wrinkled, parchment-like appearance [66]. The appearance is thought to resemble being “splashed with whitewash” [75]. Other features include erythema, telangiectasia, purpura, bullae, and hemorrhage [76].

### 6.3. Treatment

There are limited data regarding treatment modalities for hypopigmentation in EGLS [74]. The British Association of Dermatologists’ guidelines for management of lichen sclerosus recommends the use of high-potency topical corticosteroids to treat the underlying inflammatory process [77]. It is unclear whether the immunosuppressive effects of topical steroids prevent or improve hypopigmentation in EGLS lesions. In some cases, extended high potency steroid use may actually contribute to persistent hypopigmentation [78,79]. Topical tacrolimus has been shown to induce pigmentation in a patient with EGLS by upregulating stem cell factor (SCF) release from keratinocytes, resulting in increased proliferation and migration of melanocytes [80]. While various forms of phototherapy have long been used for extragenital LS, such as nbUVB and PUVA, UVA1(ultraviolet A1) has more recently also shown repigmentation of LS lesions in some preliminary studies [77,81]. The mechanism is likely due to an upregulation of alpha-melanocyte stimulating hormone as a protective mechanism to reduce UV radiation-induced DNA damage [82,83].

## 7. Progressive Macular Hypomelanosis

### 7.1. Etiology

Progressive macular hypomelanosis (PMH) is a chronic skin condition that is most commonly diagnosed in young women and individuals with darker skin phototypes. Studies investigating its etiology report strong evidence linking the bacterium Cutibacterium acnes (C. acnes) to the development of PMH [84]. A recent European study identified an association between the type III C. acnes lineage and PMH, which may suggest involvement of a type III-specific factor that causes hypopigmentation through disruption of melanogenesis or direct depigmentation effects. However, the underlying mechanism has yet to be elucidated [84,85,86,87,88,89,90,91]. Histology reveals diminished pigment in the affected epidermis with a normal-appearing dermis and normal melanocyte count [92]. Ultrastructural examination of lesional skin reveals immature, aggregated melanosomes in contrast to mature melanosomes in unaffected skin, suggesting alterations in melanocyte transfer [93,94]. Increased keratinocyte apoptosis and atypical distribution of tonofilaments surrounding melanosomes, which have been reported in one study, may potentially explain abnormalities in melanosome maturation and distribution [95].

### 7.2. Clinical Manifestations

PMH presents as poorly-defined hypopigmented macules or patches without scale or itch. Lesions are typically confluent and symmetric. PMH most commonly affects the anterior and posterior trunk, although lesions may occasionally extend to involve the upper extremities, neck, face, and buttocks. Inflammation is notably absent, unlike in post-inflammatory hypopigmentation. The diagnosis of PMH is usually made clinically but can be supported by Wood’s lamp examination, which can reveal orange-to-red fluorescence glow indicative of C. acnes [96].

### 7.3. Treatment

Treatment modalities for PMH aim to reduce the bacterial load of C. acnes, particularly the type III lineage. Options include phototherapy, oral tetracyclines, topical clindamycin, and benzoyl peroxide, although a small percentage of patients with PMH spontaneously resolve in mid-life [84,85,90,97]. An early clinical trial demonstrated more effective repigmentation with combination benzoyl peroxide 5% gel, clindamycin 1% lotion, and UVA phototherapy compared to fluticasone 0.05% cream with UVA phototherapy alone. These results demonstrate the importance of antibacterial therapy on repigmentation and support the proposed causal relationship between C. acnes and PMH [98]. Subsequent studies have demonstrated the efficacy and safety of nbUVB, which achieves repigmentation through stimulation of residual melanocytes and inhibition of C. acnes growth [99,100]. Compared to patients who used benzoyl peroxide and topical clindamycin, patients who completed a three-month course of nbUVB twice a week achieved higher remission rates (90% vs. 38%) in a shorter timeframe (2.5 vs. 8.5 months). Among patients who achieved remission, 6% of those in the nbUVB group relapsed (average of 10.2 months) compared to none in the antimicrobial group. The results of these trials may suggest that nbUVB appears to be the most effective treatment for PMH but runs the risk of relapse in the long term [101]. Thus, modalities combining topical antibiotics with phototherapy may be recommended to achieve quick remission while minimizing relapse rates. Future studies are needed to directly compare nbUVB monotherapy to nbUVB combination therapy with antimicrobials [102].

## 8. Inflammatory Diseases

### 8.1. Scleroderma

#### 8.1.1. Etiology

The term scleroderma represents a group of autoimmune fibrosing disorders including localized, systemic, limited cutaneous, and diffuse cutaneous variants. The pathophysiology is thought to involve Th2-driven immune production leading to an increase in profibrotic IL4, which induces the production of TGFB and collagen by fibroblasts and leads to an imbalance of angiogenic mediators, resulting in a self-perpetuating process of fibrosis and vascular injury [103]. Resulting scleroatrophic lesions with focal hyperpigmentation or hypopigmentation are thought to likely represents post -inflammatory changes [104]. Histopathological studies of pigment loss in some lesions of juvenile localized scleroderma showed a decreased number of melanocytes at the dermal–epidermal junction [105]. A possible explanation is an immune mediated loss of melanocytes, as immunostaining was skewed towards more CD3+ and CD8+ in hypopigmented lesions [105]. Although clinical findings are similar to vitiligo, loss of pigmentation is commonly seen in areas of localized sclerosis. Case reports of patients with systemic sclerosis have described diffuse depigmentation except for perifollicular and supravenous retention of pigment [106,107,108]. The exact mechanism of the dyspigmentation is unknown, but it is theorized that immune dysfunction and external factors, such as trauma, trigger an inflammatory cascade, resulting in a T-lymphocyte mediated destruction of melanocytes, as seen in vitiligo [106,107,109]. However, melanogenesis is preserved around the perifollicular skin due to the rich capillary network, resulting in perifollicular retention of pigment [106].

#### 8.1.2. Clinical Manifestations

The common pigmentary changes that occur in systemic sclerosis are generalized hyperpigmentation, hypo-and hyperpigmentation in areas of sclerosis, and a leukoderma resulting in a salt and pepper appearance. The latter is a vitiligo-like depigmentation with patches of perifollicular hyperpigmentation most commonly seen on the upper part of the trunk and distal extremities [110]. This is one of the earliest cutaneous findings of systemic sclerosis, seen in about 51–94% of patients [107].

#### 8.1.3. Treatment

There are no current therapies for management of dyspigmentation seen in scleroderma; existing treatments seek to manage complications and provide symptomatic relief of systemic symptoms [111]. A case of UV-induced repigmentation was seen in a patient with systemic scleroderma, who had extensive patches of hypopigmentation formed with perifollicular sparing. The left arm re-pigmented after preferential exposure to natural sunlight for several years [112]. Phototherapy could be beneficial to induce repigmentation in scleroderma, although a risk of hyperpigmentation exists [113]. Since the pathogenesis of scleroderma is similar to that of vitiligo, benefits of phototherapy may be due to inducing T suppressor cell activity, thereby preventing destruction of melanocytes, and stimulation of melanocyte differentiation and migration [114]. High quality studies are needed to understand efficacy of therapies targeted toward pigmentary alterations seen in scleroderma.

### 8.2. Discoid Lupus Erythematosus

#### 8.2.1. Etiology

Discoid lupus erythematosus (DLE) is the most common type of chronic cutaneous lupus erythematosus. DLE is a connective tissue disease characterized on histology by a lichenoid reaction pattern with vacuolar change, follicular plugging, and inflammatory infiltrates dominated by T helper 1 (Th1) cells. Increased plasmacytoid dendritic cell and type I interferon (IFN) signaling in DLE leads to expression of pro-inflammatory cytokines, which has been correlated with extent of disease activity [115]. Ultimately, this inflammatory cascade causes damage to basal keratinocytes and melanocytes, leading to scarring and hypopigmentation [116]. The mediators TNF, IL-1a, IL-1b, IL-6, and IL-17 have been shown to inhibit melanogenesis and may potentially be involved in the pathogenesis of DLE [65].

#### 8.2.2. Clinical Manifestations

Patients with DLE develop annular erythematous patches or plaques. As the lesions expand, patients are left with atrophic hypopigmented lesions or scarring alopecia surrounded by hyperpigmentation [117]. These lesions are typically localized to the face, ears, or scalp. Post-inflammatory hypopigmentation is another potential manifestation of DLE, as are verrucous hyperkeratotic plaques that leave behind atrophic hypopigmented scars as they heal [117].

#### 8.2.3. Treatment

Current first-line treatment for DLE consists of photoprotection and topical or intralesional corticosteroids and/or topical calcineurin inhibitors [117,118]. Pulsed dye laser has been investigated for the treatment of pigmentary changes in DLE but failed to demonstrate significant change in a randomized controlled trial [119]. Melanocyte-keratinocyte transplantation, epidermal grafting, and melanocyte grafting involve the transfer of melanocytes to hypopigmented areas. These have led to significant improvements in repigmentation in select cases, however, larger studies are needed to better define the efficacy of these treatments [116,120].

## 9. Infectious Diseases

### 9.1. Pityriasis Versicolor Alba

#### 9.1.1. Etiology

Pityriasis versicolor (PV), also known as tinea versicolor, is a superficial fungal infection caused by conversion of *Malassezia* from its yeast to mycelial form [98]. The hypopigmented variant of PV is known as pityriasis versicolor alba (PVa) [121]. Electron microscopy of PVa lesions reveal damaged melanocytes and decreased number of melanosomes in keratinocytes, suggesting impairment in melanosome transfer [122]. The underlying etiology is thought to involve oxidation of unsaturated fatty acids by *Malassezia*, leading to production of lipoperoxides and dicarboxylic acids, such as azelaic acid. These byproducts have been shown to competitively inhibit tyrosinase in vitro, thereby decreasing melanin synthesis [121,123,124,125]. Conversion of tryptophan into the indole compound malassezin, which induces melanocyte apoptosis, may also play a role [126].

#### 9.1.2. Clinical Manifestations

Individuals with PVa present with multiple, asymptomatic, and round macules/patches of hypo- or depigmented skin with fine scale. The lesions commonly become confluent, forming a larger patch with irregular borders. Areas enriched in sebaceous glands, such as the trunk, upper arms, and face, are most affected. The higher prevalence in teenagers and young adults is thought to be due to increased sebum production in these populations, allowing for growth of most *Malassezia* species [121,127]. Most diagnoses are made clinically, but light microscopy of skin scrapings prepared with potassium hydroxide (KOH) can be used for definitive diagnosis [127].

#### 9.1.3. Treatment

Antifungal therapies (halprogin, zinc pyrithione, tolciclate, ciclopirox olamine, terbinafine, and azoles) constitute the primary treatment for PV. Pathogen eradication addresses the root cause of hypopigmentation through removal of fungal metabolites produced by *Malessezia* [128,129]. Although topical treatments are inexpensive and effective when applied correctly, oral treatments may be warranted in cases where topical agents have failed or in those involving larger body surface area. Clinical trials have demonstrated efficacy of oral fluconazole in a variety of dosing regimens, including 150 mg/week for 4 weeks, 300 mg/week for 2–4 weeks, 300 mg every 2 weeks for 4 weeks, and a single 400 mg dose [130]. A 2019 randomized controlled trial compared the following treatment arms: fluconazole 300 mg per week and 2% ketoconazole foam twice a week for two weeks, itraconazole 200 mg daily for one week, and ketoconazole 2% foam daily for two weeks. Their results showed that oral fluconazole plus topical ketoconazole was most effective, with a mycological cure rate of 62.4% compared to 35.3% and 37.5% for the other regimens, respectively [131]. Ultraviolet therapy following antimycotic treatment has been shown to improve repigmentation via melanogenesis induction [122].

### 9.2. Leucoderma Syphiliticum (LSy)

#### 9.2.1. Etiology

Syphilis is a sexually transmitted infection caused by the spirochete *Treponema pallidum*. LSy is considered a manifestation of secondary syphilis and encompasses the hypopigmented lesions found in patients with this disease. In a number of LSy cases, the hypopigmented lesions developed on previously unaffected skin [132,133], with histology revealing treponemes around vessels and nerve fibers and absence of melanin in the epidermis [132,133,134]. In other cases, the rash was found to develop in areas of prior syphilitic lesions, without evidence of treponemal infection on biopsy [133]. A case report of a 35-year-old male with secondary stage syphilis demonstrated plasmocytic inflammatory infiltrates in the papillary dermis, epidermal atrophy, and pigmentary incontinence [135].

#### 9.2.2. Clinical Manifestations

Very few case reports of LSy have been published, and those that have been published are highly diverse in presentation. Lesions are typically hypopigmented with a mottled appearance, round, non-scaling, and slightly raised. Older case reports note involvement of the cervical region as a classic feature; however, involvement of the trunk, face, and extremities have also been documented [134,136,137]. More recently, reports of LS favor the upper extremities, hands, genitals, and trunk [132,135].

#### 9.2.3. Treatment

Early case reports remarked on the longevity of LSy lesions, which were noted to last years without treatment response [136,137]. In more recent literature, the hypopigmented lesions of LSy demonstrate good response to penicillin treatment, which leads to repigmentation within weeks to months in parallel with decreased VDRL titers [132,134]. The effectiveness of penicillin may suggest active treponemal infection as a cause of persistent hypopigmentation; however, the mechanism underlying hypopigmentation of prior syphilitic lesions remains poorly understood.

### 9.3. Acrodermatitis Chronica Atrophicans

#### 9.3.1. Etiology

Acrodermatitis chronica atrophicans (ACA) is a late manifestation of chronic Lyme disease due to infection with the spirochete *Borrelia afzelii*. The prevalence of ACA is up to 10% in patients with Lyme disease in Europe [138]. Failure of pathogen elimination is thought to play a role in the development of ACA, as suggested by the persistence of *B. afzelii* in cutaneous lesions years after initial infection. The downregulation of major histocompatibility complex class II molecules on Langerhans cells is thought to be an indicator of a poor immune response and pathogen eradication [139,140]. The spirochete’s affinity for extracellular matrix proteins and collagen causes degradation of connective tissues, ultimately leading to fibrosis and atrophy [141,142,143,144]. On histopathology, ACA is characterized by an early dermal perivascular infiltrate composed of lymphocytes and plasma cells, with telangiectasia and mild epidermal atrophy. Late stages are characterized by more advanced epidermal and dermal atrophy, orthokeratosis, periadnexal fibrosis, and occasional histiocytes or mast cells [10,92]. The predominance of CD3+ and CD4+ cells in the dermal infiltrate suggest that the development of ACA is mediated by a chronic T-cell-mediated immune reaction [138,145]. Necrosis of basilar keratinocytes can also be seen, which may potentially contribute to development of hypopigmentation; however, the etiology of hypopigmentation in ACA has not been well-studied [146].

#### 9.3.2. Clinical Manifestations

ACA is classically described in two stages. The initial inflammatory stage is characterized by red to violaceous plaques and nodules involving the extensor surfaces of distal extremities. The underlying skin is typically swollen. Over months to years, this gives way to the second stage, characterized by chronic hypopigmentation and thin shiny skin. Hyperpigmentation, prominent blood vessels, fibrous nodules, pain, pruritus, peripheral neuropathy, and scale may also be present [92]. The diagnosis relies on clinical signs in addition to histopathology and serologic testing for *Borrelia* antibodies.

#### 9.3.3. Treatment

ACA lesions do not resolve spontaneously. The treatment is systemic antibiotic therapy with amoxicillin, doxycycline, penicillin G, ceftriaxone, or cefotaxime to achieve spirochete elimination, as the pathogenicity of *B. afzelii* underlies the clinical manifestations. Antibiotics should be taken for four weeks, as shorter durations have proved less efficacious [147,148]. As ACA progresses, the condition becomes more treatment-resistant, and chronic skin changes may only be partially reversible. In one study, ceftriaxone therapy for 28 days led to partial-to-complete resolution of cutaneous lesions; however, skin atrophy and bluish red discoloration persisted despite treatment [149]. The existing literature does not distinguish between treatment of hypopigmented versus hyperpigmented lesions, and future studies are needed to evaluate treatments for ACA-associated hypopigmentation.

## 10. Mycosis Fungoides (MF)

### 10.1. Etiology

MF is the most common type of cutaneous T-cell lymphoma in both adults and children. The etiology of MF is not well understood, but hypotheses have been proposed associating the disease with immunosuppressed states, infectious agents, chronic exposure to allergens or carcinogens, or abnormalities in genetic and cytokine profiles [150,151,152,153,154,155]. Histopathologically, the classic subtype of MF is characterized by an epidermotropic infiltrate of irregular “cerebriform” T lymphocytes with a CD4+ predominance. Many variants of MF have been described, including the hypopigmented, pustular, bullous, verrucous, and hyperkeratotic subtypes [156,157,158,159]. The purely hypopigmented variant, HMF, is uncommon overall, though is more frequently reported in younger populations and has been described almost exclusively in skin of color [160,161,162]. In contrast to the classic form, HMF is characterized on histology by epidermotropism of haloed CD8+ lymphocytes dominated by a T-suppressor lineage, in the absence of dense dermal infiltrate. Upregulation of Th1 cytokines, most notably TNF-α, has been associated with an antitumor effect against progression of infiltrative disease [163,164]. The cytotoxic, non-neoplastic predominance is thought to inhibit melanogenesis through the direct destruction of melanocytes. Another explanation for hypopigmentation involves induced defects in melanosome transfer, as evidenced by a decreased number of melanosomes seen within keratinocytes and a normal density of melanosomes within melanocytes [165].

### 10.2. Clinical Manifestations

The progression of classic MF is described in three phases (patch, plaque, tumor) that evolves over years, although the lesions frequently co-exist with each other. Over 90% of patients with early MF do not progress to tumor stage, while other individuals may develop tumors without having first developed the patches or plaques. In contrast, HMF is characterized by hypopigmented or achromic patches or plaques of varying sizes [155,166]. The lesions may be accompanied by variable pruritus, telangiectasia, or atrophy [167]. HMF is usually diagnosed in the patch stage and is associated with a better prognosis than classic MF, as it typically does not progress beyond stage IB [161,167].

### 10.3. Treatment

Phototherapy is a mainstay of HMF treatment and involves PUVA and nbUVB irradiation [167,168]. PUVA induces melanogenesis through activation of TRPV1 and TRPA1 channels in melanocytes [169]. It also causes apoptosis of malignant cells via induction of DNA damage [157]. nbUVB is a potent enhancer of pro-inflammatory cytokine production by keratinocytes and epidermal antigen-presenting cells, effectively suppressing malignant cell proliferation [161,170,171]. These mechanisms ultimately reduce the antitumor response and lead to repigmentation in most cases. Other case reports have also demonstrated repigmentation with topical nitrogen mustard, reversing the defect in melanosomes transfer to keratinocytes through an unknown mechanism [160,165,172].

## 11. Iatrogenic Hypopigmentation

### 11.1. Etiology

Skin resurfacing tools, such as lasers, chemical peels, and dermabrasion, carry a risk of hypopigmentation [173]. Incidence estimates vary widely, with the loss of pigmentation having been reported to occur in between 1% and 20% of patients. Hypopigmentation after skin resurfacing is postulated to result from a suppression of melanogenesis rather than destruction of melanocytes and may involve the disruption of signaling processes, resulting in a downregulation of proopiomelanocortin and its associated down-stream effectors [174,175]. Ex vivo scalp skin biopsies following treatment with the ruby laser, a method for removal of unwanted body hair, revealed decreased tyrosinase activity, potentially due to heat or disruption of enzyme activity, in addition to the predicted reduction in melanin content. Another case report of delayed hypopigmentation following carbon dioxide laser resurfacing revealed a decrease in epidermal melanin without reduction in the number of melanocytes, similar to what is seen after phenol bleaching and dermabrasion [176,177,178]. In addition to impaired melanogenesis, other mechanistic explanations for resurfacing-induced hypopigmentation include disruptions in the basement membrane and collagen [174].

Another common iatrogenic cause of hypopigmentation is use of high-dose topical or intralesional corticosteroids [78,79]. These can inhibit prostaglandin and cytokine production, which are necessary mediators of melanocyte function [179,180]. Development of hypopigmented linear streaks after topical steroid application, while uncommon, may occur due to lymphatic or venous spread of corticosteroid crystals, causing pigmentary changes on overlying skin [180].

Increased mask use, seen after the COVID-19 pandemic, has resulted in multiple dermatoses and worsening of underlying inflammatory skin conditions (i.e., seborrheic dermatitis, atopic dermatitis, and psoriasis) [181,182,183]. In mask-covered areas, this pro-inflammatory microenvironment and local disruption of skin barrier function can potentially lead to pigmentary changes [182,184]. In patients with psoriasis or other inflammatory dermatoses, friction from masks or other physical trauma can induce development of new lesions in uninvolved skin via the Koebner phenomenon, which can indirectly cause PIH [1,182]. Mask wearing is also thought to disrupt the healthy skin microbiome, selecting for preferential growth of pathogenic strains, such as *C. acnes*, which further contributes to cutaneous inflammation via activation of innate immunity [185]. In addition, non-CE (European conformity mark) masks, not subject to rigorous material and safety testing, have shown to result in increased incidence of irritant contact dermatitis [186].

### 11.2. Clinical Manifestations

Hypopigmented macules or patches are seen in the treatment area where the procedure (i.e., laser, chemical peels, dermabrasion) was performed. For example, dyspigmentation caused by laser is observed as white macules that match the size and shape of the laser spot [1].

### 11.3. Treatment

Treatment can range from conservative management (silicone sheeting and massage) to invasive (melanocyte-keratinocyte transplantation) [175,187]. Silicone sheeting and massage are simple to perform and affordable, but do not have strong evidence supporting their efficacy [175,187]. Melanocyte-keratinocyte transplantation is an invasive surgical technique involving the harvesting of donor site skin, which is digested with trypsin, made into a cell suspension, and applied to the hypopigmented area after microdermabrasion [175,188]. This can be very costly, and very few centers offer this resource. Intermediate options include excisional scar revision, split thickness grafting, laser therapy, medical tattooing, and microneedling [175,187]. Of these options, a combination laser treatment with topical prostaglandin analogue with or without topical retinoid was found to result in superior repigmentation in one study [175]. Other combination techniques include laser-assisted chemabrasion, which consists of a TCA peel followed by carbon dioxide (CO_2_) laser, then dermabrasion [173].

With regards to laser therapy, a popular laser choice in dermatology is ND:YAG (neodymium-doped yttrium aluminum garnet), which can be used for treatment of hyperpigmentation through destruction of melanin granules [189]. Excessive reduction of epidermal melanin can sometimes inadvertently lead to hypopigmentation. Two studies reported reversal of Q-switched laser induced hypopigmentation with nbUVB phototherapy [190,191]. Benefits are likely due to stimulation of melanocytes and increased melanin synthesis. A different study reported the reversal of Q-switched induced hypopigmentation with excimer laser due to its activating effect on melanocytes [192]. Non-fractional CO_2_ laser induced hypopigmentation was corrected with three sessions of fractionated CO_2_ laser in one case series [193]. In addition, PUVA was found to be beneficial for repigmentation after CO_2_ laser induced hypopigmentation, resulting in moderate to excellent repigmentation in 71% of the treated patients [174].

## 12. Discussion

Mechanisms of hypopigmentation can result from multiple mechanisms, including decreased melanin production, impaired transfer of melanosomes to keratinocytes, and melanocyte destruction/dysfunction. Hypopigmentation seen in PLC, EGLS, scleroderma, and MF mostly result from the destruction of melanocytes, and as a result, have often been observed to show induction of repigmentation with phototherapy (PUVA, nbUVA). LS also results from the destruction of melanocytes; yet, it has been reported to respond more favorably to topical steroids, tacrolimus, and excimer laser. PA, PMH, DLE, PVA, and other infectious causes of PIH responded well to therapy targeted toward resolving the infection and/or dampening of the inflammatory response with either steroids, calcineurin inhibitors, antibiotics, or antifungals. Some showed additional benefit from laser or phototherapy. Although steroids are the mainstay of therapy in many of these conditions, they carry an additional risk of steroid induced hypopigmentation with onset that may be delayed for up to 4 months.

In general, dyspigmentation can lead to great cosmetic and psychosocial morbidity, impacting quality of life in patients. Various scoring instruments have been developed to better describe hyperpigmentation [194,195,196], however, there is lack of validated tools to measure PIH specifically. Although scoring systems exist to characterize severity of vitiligo lesions [197], they are unlikely to capture the subtlety of the hypopigmented lesions seen in PIH. In addition, the extent, stage, and progression of disease is dissimilar between vitiligo and PIH, which limits the applicability of these tools. An unmet need exists for an inclusive, standardized outcome measure to assess PIH.

**Figure 1:**
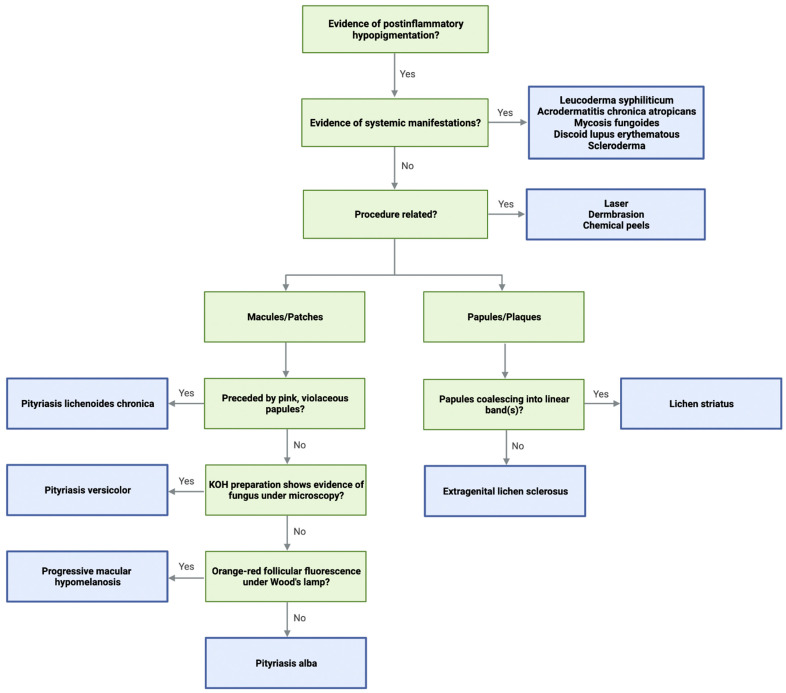
Diagnostic flow chart for post-inflammatory hypopigmentation.

Based upon our discussion of etiology, clinical manifestations, and current treatment options for PIH, we built a diagnostic algorithm to assist physicians in diagnosing PIH (Figure 1). Algorithms for the diagnosis of hypopigmentation disorders have been previously published [198,199,200]; however, they do not distinguish between the various diagnoses of PIH. To our knowledge, this is the first publication to guide physicians and provide a systematic diagnostic approach for PIH.

### Limitation/Bias

A limitation is that the exact mechanisms of hypopigmentation in many conditions discussed in this manuscript have not been fully elucidated. Clinical manifestations may also differ across various skin phototypes, further complicating algorithmic approaches to diagnosis and treatment. Additionally, reviewed publications on therapeutic options for PIH consisted of variable treatment durations and scheduling, leading to potential heterogeneity and limiting accurate comparisons. Literature on pigmentation-specific therapies often is limited to data from case reports and recurrence of PIH after treatment cessation is often not documented. Lastly, the nonsystematic nature of this review introduces bias as selection of articles was based on authors’ professional expertise.

## 13. Conclusions

PIH is the end result of a wide range of inflammatory and infectious dermatoses. The exact pathogenesis of PIH is unknown, although various mechanisms have been proposed in the literature. Pigmentary changes secondary to cutaneous inflammation are also dependent on the individual’s skin phototype and individual predisposition to dyschromia. Although therapeutic advances have been made to address many of the underlying diseases causing PIH, specific therapies targeting resultant dyschromia are limited. Well-designed studies with standardized outcome measures to assess safety and efficacy will assist in the identification of targeted therapies for PIH in the future.

## Data Availability

Not applicable.

## Abbreviations

Pityriasis alba (PA); post-inflammatory hypopigmentation (PIH); MITF (microphthalmia-associated transcription factor); psoralens plus UVA (PUVA); pityriasis lichenoides chronica (PLC); pityriasis licehnoides et varioliformis acuta (PLEVA); basic fibroblast growth factor (bFGF); hypopigmented MF (HMF); extragenital lichen sclerosus (EGLS); lichen sclerosus (LS); stem cell factor (SCF); nbUVB (narrowband ultraviolet B); UVA1(ultraviolet A1); UV (ultraviolet); progressive macular hypomelanosis (PMH); Cutibacterium acnes (C. acnes); UVA (ultraviolet A); discoid lupus erythematosus (DLE); T helper 1 (Th1); pityriasis versicolor (PV); pityriasis versicolor alba (PVa); leucoderma Syphiliticum (LSy); acrodermatitis chronica atrophicans (ACA); mycosis fungoides (MF); tumor necrosis factor-α (TNF- α); carbon dioxide (CO_2_); ND: YAG (neodymium-doped yttrium aluminium garnet).
